# Combined killing of cancer cells and cross presentation of tumor antigen by Vγ9Vδ2 T cells

**DOI:** 10.1186/2051-1426-3-S2-P327

**Published:** 2015-11-04

**Authors:** Gitte Holmen Olofsson, Manja Idorn, Ramona Schenker, Elfriede Nössner, Reno Debets, Bernhard Moser, Özcan Met, Per thor Straten

**Affiliations:** 1 Centre for Cancer Immune Therapy, Dept. of Hematology, Copenhagen University Hospital, Herlev, Denmark; 2 Helmholtz Zentrum München, Germany Research Center for Environmental Health, Institute for Molecular Immunology, Münich, Germany; 3 Laboratory of Experimental Tumor Immunology, Erasmus MC Cancer Institute, Rotterdam, Netherlands; 4 Department of Infection, Immunity & Biochemistry, School of Medicine, Cardiff, UK; 5 Centre for Cancer Immune Therapy (CCIT), Copenhagen University Hospital Herlev, Herlev, Denmark

## 

The human Vγ9Vδ2 T cells are a unique T cell type, and recent studies of the biology of Vγ9Vδ2 T cells emphasize the potential exploitation of these cells in immunotherapy of cancer. Vγ9Vδ2 T cells exhibit dual functionality in that they are both antigen presenting cells (APC) and cytotoxic towards cancer cells. We show that Vγ9Vδ2 T cells can kill cancer cell lines from various cancer types such as leukemia, melanoma, prostate-, and breast cancer, with a significantly increased killing upon treatment of the cancer cells with Zoledronic acid. In addition, we show that Vγ9Vδ2 T cells take up tumor antigens gp100 and MART-1 (long peptide and recombinant protein, respectively), and process these antigens for presentation of class I restricted peptides in the context of the HLA-A02.01 molecule, to be recognized by peptide specific cytotoxic CD8 T cells. Moreover, we show that specific inhibition of the proteasome by lactacystin impair recognition by peptide specific CD8 T cells, strongly suggesting proteasome involvement in presentation of the relevant class I restricted peptides. The dual functions; killing and antigen presentation combined with the ease of expanding Vγ9Vδ2 T cells in vitro from peripheral blood lymphocytes to billions of cells, makes Vγ9Vδ2 T cells attractive vehicles for adoptive cell therapy (ACT) in cancer therapy. Thus, Vγ9Vδ2 T cells are broadly tumor specific killers, that concurrently could induce or support tumor specific αβ-T cell responses.

